# Pneumococcal polysaccharide vaccination in adults undergoing immunosuppressive treatment for inflammatory diseases – a longitudinal study

**DOI:** 10.1186/s13075-015-0663-9

**Published:** 2015-06-06

**Authors:** Lara Fischer, Patricia Francis Gerstel, Antoine Poncet, Claire-Anne Siegrist, Emmanuel Laffitte, Cem Gabay, Joerg Dieter Seebach, Camillo Ribi

**Affiliations:** Division of Clinical Immunology and Allergy, Department of Medical Specialties, University Hospital and Faculty of Medicine, Geneva, Switzerland; Division of Dermatology, Department of Medical Specialties, University Hospital and Faculty of Medicine, Geneva, Switzerland; Division of Clinical Epidemiology, Department of Health and Community Medicine, University Hospital and Faculty of Medicine, Geneva, Switzerland; Center for Vaccinology, University Hospital and Faculty of Medicine, Geneva, Switzerland; Division of Rheumatology, Department of Medical Specialties, University Hospital and Faculty of Medicine, Geneva, Switzerland; Division of Immunology and Allergy, Lausanne University Hospital CHUV, Lausanne, Switzerland

## Abstract

**Introduction:**

Patients undergoing immunosuppressive therapy are at increased risk of infection. Community-acquired pneumonia and invasive pneumococcal disease account for substantial morbidity and mortality in this population and may be prevented by vaccination. Ideally, immunization to pneumococcal antigens should take place before the start of immunosuppressive treatment. Often, however, the treatment cannot be delayed. Little is known about the efficacy of pneumococcal vaccines during immunosuppressive treatment. The objectives of this study were to determine the percentage of vaccine-naïve, immunosuppressed adults with inflammatory diseases seroprotected against *Streptococcus pneumoniae* and to assess factors associated with the immunogenicity, clinical impact and safety of 23-valent pneumococcal polysaccharide vaccine (PPV) in seronegative subjects.

**Methods:**

This observational study included patients 18 years of age and older who were receiving prednisone ≥20 mg/day or other immunosuppressive drugs. Exclusion criteria were PPV administration in the previous 5 years, intravenous immunoglobulins and pregnancy. Serum immunoglobulin G (IgG) antibody levels against six pneumococcal serotypes were measured. Seropositivity was defined as IgG of 0.5 μg/ml or greater for at least four of six serotypes. Seronegative patients received PPV, and seropositive patients were included as a comparison group. Vaccine response and tolerance were assessed after 4–8 weeks. Disease activity was evaluated on the basis of the Physician Global Assessment scores. Serology was repeated after 1 year, and information on any kind of infection needing medical attention was collected. Outcomes were the proportion of seropositivity and infections between vaccinated and unvaccinated patients.

**Results:**

Of 201 included patients, 35 received high-dose corticosteroids and 181 were given immunosuppressive drugs. Baseline seronegativity in 60 (30 %) patients was associated with corticotherapy and lower total IgG. After PPV, disease activity remained unchanged or decreased in 81 % of patients, and 87 % became seropositive. After 1 year, 67 % of vaccinated compared with 90 % of observed patients were seropositive (*p* < 0.001), whereas the rate of infections did not differ between groups. Those still taking prednisone ≥10 mg/day tended to have poorer serological responses and had significantly more infections.

**Conclusions:**

PPV was safe and moderately effective based on serological response. Seropositivity to pneumococcal antigens significantly reduced the risk of infections. Sustained high-dose corticosteroids were associated with poor vaccine response and more infections.

## Introduction

Infections cause substantial morbidity and mortality among patients with immune-mediated inflammatory disorders [1, 2]. In rheumatoid arthritis (RA) and systemic lupus erythematosus (SLE), pneumonia accounts for up to 25 % of fatalities [3]. One risk factor for infection in these patients is the frequent use of immunosuppressive therapies. Prednisone has been associated with pneumonia [4, 5], and cyclophosphamide, azathioprine and newer biologic agents such as tumor necrosis factor (TNF)-α blockers have been shown to confer an increased risk of infection [6–9]. Another risk factor arises from immune defects associated with the inflammatory disorder itself [1, 10].

Guidelines recommend the administration of pneumococcal vaccines to patients from receiving immunosuppressive treatment [11–14]. However, only a minority of patients are vaccinated against *Streptococcus pneumoniae* [15, 16]. Several factors may account for this low rate of vaccination. First, there are doubts concerning the immunogenicity of vaccines in patients undergoing immunosuppressive therapy [17, 18]. The suppression of lymphocyte proliferation and function might control the underlying condition but simultaneously lead to impaired antigen responses. Second, there is lack of proof regarding the efficacy of pneumococcal polysaccharide vaccine (PPV) in preventing invasive pneumococcal disease (IPD), pneumonia and mortality for patients with chronic illnesses [19]. There are also concerns about the duration of vaccine response in these patients. Indeed, PPV is based solely on capsular polysaccharides, which behave as T-cell-independent antigens and induce a limited humoral response without generation of memory B cells. It has been shown that post-vaccine antibodies wane faster in patients under immunosuppressive therapies than in healthy individuals [20, 21]. Last, there have been case reports of a causal relationship between pneumococcal vaccination and the clinical onset or flare-ups of immune-mediated inflammatory disorders, in particular in autoinflammatory conditions [22]. However, several prospective studies have shown that patients may be immunized without exacerbation of the underlying inflammatory disease [11, 23, 24]. There are limited data on the utility of pneumococcal vaccines in adult patients undergoing immunosuppressive therapy for immune-mediated inflammatory disorders. A retrospective study in patients with RA treated with methotrexate found a significantly lower incidence of pneumonia over the previous 10 years in patients having received PPV compared with those never vaccinated [25].

We conducted a prospective, single-center, observational study to address the following issues: What is the proportion of seronegativity to *S. pneumoniae* in a population of patients with immune-mediated inflammatory disorders under immunosuppressive treatment not vaccinated with PPV in the previous 5 years? Are there factors associated with seronegativity to pneumococcal antigens? Does immunization with PPV induce disease flare-up? Does PPV elicit sufficient responses to pneumococcal antigens under immunosuppressive therapy? How do antibody levels to *S. pneumoniae* evolve over time in patients vaccinated and observed?

We also wanted to assess the rate of clinically relevant infection over the course of 1 year with respect to pneumococcal serology and examine whether other factors were associated with a higher risk of infection.

## Methods

### Patients

We conducted an observational study in an outpatient setting. Between November 2008 and October 2011, adult patients followed at the University Hospital of Geneva were screened to participate. Inclusion criteria were a minimum age of 18 years, a diagnosis of immune-mediated inflammatory disorder and ongoing treatment with high-dose systemic corticosteroids (≥20 mg/day for ≥1 month) and/or immunosuppressive drugs (classical immunosuppressive drugs and/or biologic immunomodulatory agents). Exclusion criteria were vaccination with PPV in the previous 5 years, previous treatment with intravenous immunoglobulins and pregnancy. None of the patients was exposed to previous conjugate pneumococcal vaccine, which was limited to pediatric patients at the time of the study. The study was approved by the local ethics review board of the University Hospitals Geneva (Commission cantonale d’éthique de la recherche), and all patients gave their written informed consent to participate. Inclusion started in the Division of Clinical Immunology as a pilot study and was then extended to the divisions of rheumatology and dermatology.

### Assessment

Patients were assessed at baseline, 4–8 weeks after immunization and after 1 year. Clinical data included the nature of the underlying diseases, type and duration of immunomodulatory treatments, previous influenza and pneumococcal vaccination, disease activity and infections. Inflammatory disorders were categorized into five groups: (1) RA and spondylarthropathies, (2) psoriasis and psoriatic arthritis, (3) connective tissue diseases, (4) systemic vasculitis and (5) others. The last group included patients with rare inflammatory diseases that could not be classified into a larger group, such as bullous skin diseases and autoinflammatory syndromes. Information on previous vaccines was gathered by reviewing the certificate of vaccination or, if that was not available, by reviewing the clinical charts and by patient recall. Disease activity was assessed by using the Physician Global Assessment (PGA) score with a 4-point scale, ranging from 0 to 3 (0 = inactive disease, 1 = low disease activity, 2 = active disease and 3 = very active disease). PGA was scored at baseline, 4–8 weeks after immunization and at the end of follow-up. Infections were defined as infectious events needing medical attention, regardless of severity. This information was collected retrospectively at baseline and at the end of the study with a patient questionnaire (patients were asked about symptoms, diagnosis and medicines prescribed) by reviewing the electronic medical records and by contacting the primary care physician when needed. Safety aspects were based on assessment of disease activity 4–8 weeks after the immunization and oral questioning about systemic vaccine reactions. Changes in immunosuppressive treatment were assessed at the end of follow-up. Serum was sampled at each assessment.

### Pneumococcal serology

Baseline serologies were performed in real time to allow decisions to be made regarding either vaccination or observation. Levels of specific immunoglobulin G (IgG) antibody against three pneumococcal serotypes (14, 19F and 23F) and three supplementary serotypes (1, 5, 7F or 9N, 11A and 17F) were determined by enzyme-linked immunosorbent assay (ELISA) according to the World Health Organization (WHO) consensus protocol [26], which includes serum preadsorption with pneumococcal cell wall- and serotype 22F polysaccharides and uses 89-SF as a reference for antibody quantification. Supplementary serotypes 1, 5 and 7F were replaced in 2011 by 9N, 11A and 17F because the 13-valent conjugated vaccine had become available, although it was not used in this study. Patients with specific IgG levels ≥0.5 μg/ml to at least four of the six tested serotypes were empirically considered as having sufficient antibodies, as not requiring immunization and defined as “seropositive.” Positive vaccine response was defined by reaching seropositivity 4–8 weeks after PPV. Patients having received PPV off-protocol or treated with B-cell-depleting therapies within 6 months of the vaccine were not assessed for vaccine response.

### Total serum immunoglobulins

Serum IgG, IgA and IgM were measured with an IMMAGE nephelometer (Beckman Coulter, Nyon, Switzerland) and specific anti-sera against heavy chains γ, α and μ, respectively. Normal values were established with sera of healthy blood donors.

### Vaccination procedure

Seronegative patients were offered the 23-valent PPV (PNEUMOVAX 23; Merck, West Point, PA, USA), which was the sole pneumococcal vaccine licensed for adults at the time of the study. PPV contains 25 μg of pneumococcal capsular polysaccharides of 23 serotypes (1, 2, 3, 4, 5, 6B, 7F, 8, 9N, 9V, 10A, 11A, 12F, 14, 15B, 17F, 18C, 19A, 19F, 20, 22F, 23F and 33F) [27]. The vaccine was given intramuscularly.

### Objectives

The primary study objectives were to compare the proportion of seropositives at baseline and after 1 year in vaccinated and observed patients. The secondary objectives were the assessment of changes in disease activity before and after immunization and to compare the rate of infections in the respective groups.

### Statistical analysis

Data were analyzed using the STATA statistical software package (version 13; StataCorp, College Station, TX, USA). Normally distributed data were described by means with standard deviations and skewed data by medians and interquartile ranges (IQRs). Non-parametric tests for significance (Wilcoxon rank-sum test for continuous data, *χ*^2^ test or Fisher’s exact test for categorical data) were conducted where appropriate. As IgG titers for individual serotypes relied on several real-time assessments and had censored values in the lower and upper ranges, we used reverse cumulative distribution curves [28] and the paired Prentice–Wilcoxon test [29]. Determinants for serological response at baseline were assessed with logistic regression analysis and for individual serotypes using a lognormal model. Predictors of vaccine response and infections were assessed using univariate log-binomial regression models. A *p*-value less than 0.05 was considered statistically significant in all analyses.

## Results

**Fig. 1:**
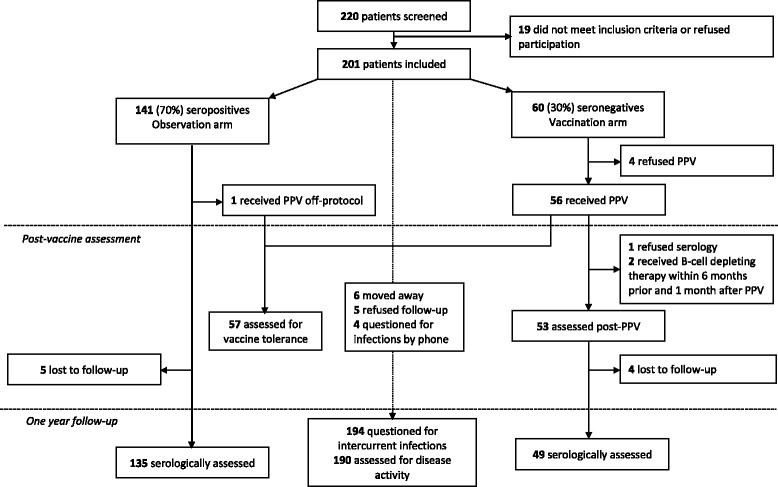
Flowchart of the study. *PPV* pneumococcal polysaccharide vaccine

Two hundred one patients were included in the study (Fig. 1). Sixty patients met the criteria for PPV administration. Four patients refused vaccination. Fifty-seven received PPV and were assessed for tolerance, including one patient in the observation group who received the vaccine off-protocol. Vaccine response was assessed in 53 patients. One hundred ninety (95 %) patients were followed for a median (IQR) of 384 (364–421) days. Eleven patients could not be assessed: six moved away and five refused the follow-up visit, with four of them contacted by telephone and questioned for infectious events. Long-term serological response was assessed in 184 patients, of whom 49 had been vaccinated and 135 observed.

### Baseline characteristics

The demographics, baseline characteristics and treatment of the 201 included patients are shown in Table 1. One hundred eighty-one patients (90 %) were treated with immunosuppressive drugs, and twenty patients (10 %) had high-dose corticosteroids only. Systemic corticosteroids were used in 96 % of patients with vasculitis and in 4 % of patients with psoriasis (*p* < 0.001). Cytotoxic drugs were used in 75 % of patients with vasculitis and 35 % with psoriasis (*p* = 0.001). Biologic immunosuppressants were used as monotherapy in 34 patients with psoriasis (65 %), 20 with RA and/or spondylarthropathies (36 %), 4 with connective tissue diseases and miscellaneous conditions, and none with vasculitis. Seventeen patients (9 %) had received rituximab at a median (IQR) of 337 (14–1652) days before inclusion. One hundred seventeen patients (60 %) received in 2009 a split seasonal influenza vaccine, one hundred five (54 %) were given an AS03-adjuvanted pandemic influenza vaccine and one hundred twelve (57 %) received a split seasonal influenza vaccine in 2010. According to enrollment criteria, none of the patients had received pneumococcal vaccines in the previous 5 years. Of the 190 patients in which written information on previous immunizations was available, none had ever received a pneumococcal vaccine.

**Table 1:** Demographic, disease and treatment characteristics of 201 patients at inclusion and comparison between groups according to baseline pneumococcal serology

Characteristics	All (*N* = 201)	Vaccination group^a^ (*n* = 60)	Observation group^b^ (*n* = 141)	*p*-Value
Median age (IQR), yr	53.9 (43.3–65.7)	58.6 (37.0–71.9)	53.1 (42.6–61.9)	NS
Male/female, *n*	84/117	22/38	62/79	NS
Disease type				
Psoriasis, *n* (%)	52 (26)	8 (13)	44 (31)	0.03
Rheumatoid arthritis, *n* (%)	41 (20)	13 (22)	28 (20)	NS
Spondylarthropathies, *n* (%)	15 (7)	4 (7)	11 (8)	NS
Connective tissue diseases, *n* (%)	31 (15)^c^	11 (18)	20 (14)	NS
Systemic vasculitis, *n* (%)	24 (12)^d^	12 (20)	12 (9)	0.03
Miscellaneous inflammatory conditions, *n* (%)	38 (19)^e^	12 (20)	26 (18)	NS
Disease activity according to PGA				0.019
Inactive, *n* (%)	28 (14)	2 (3)	26 (18)	
Moderately active, *n* (%)	120 (60)	44 (73)	76 (54)	
Active, *n* (%)	44 (22)	12 (20)	32 (23)	
Very active, *n* (%)	9 (5)	2 (3)	7 (5)	
Treatment				
Systemic corticosteroids, *n* (%)	91 (45)	41 (68)	50 (36)	<0.001
Daily prednisone dose, median (IQR), mg	10 (5–30)	10 (5–30)	10 (7.5–27.5)	NS
Prednisone ≥20 mg/day, *n* (%)	35 (17)	16 (27)	19 (14)	0.02
Immunosuppressant, *n* (%)	181 (90)^f^	50 (83)	131 (93)	NS
Other DMARDs, *n* (%)	25 (12)^g^	10 (17)	15 (11)	NS
Previous rituximab treatment, *n* (%)	17 (9)	5 (8)	12 (9)	NS
Treatment duration, median (IQR), yr	2 (0–6)	2 (0–6)	2 (0–6)	NS
Total serum IgG, median (IQR), g/L	10.8 (8.6–13.2)	9.0 (7.5–12.1)	11.2 (9–13.4)	0.003
Total serum IgA, median (IQR), g/L	2.1 (1.6–3.1)	2.1 (1.3–3.0)	2.3 (1.6–3.2)	NS
Total serum IgM, median (IQR), g/L	0.9 (0.7–1.4)	0.8 (0.6–1.2)	1.0 (0.7–1.4)	NS

*Abbreviations*: *DMARD* disease-modifying anti-rheumatic drug, *Ig* immunoglobulin, *IQR* interquartile range, *NS* not significant, *PGA* Physician Global Assessment. *p*-Values were obtained by *χ* ^2^ and Mann–Whitney *U* tests

^a^IgG ≥0.5 μg/ml for fewer than four of six serotypes

^b^IgG ≥0.5 μg/ml for four or more of six serotypes

^c^Systemic lupus erythematosus (*n* = 13), dermatomyositis (*n* = 5), mixed connective tissue disease (*n* = 4), polymyositis (*n* = 3), anti-synthetase syndrome (*n* = 3), undifferentiated connective tissue disease (*n* = 2), systemic sclerosis (*n* = 1)

^d^Giant cell arteritis (*n* = 7), granulomatosis with polyangiitis (*n* = 6), primary angiitis of the central nervous system (*n* = 5), microscopic polyangiitis (*n* = 4), Takayasu arteritis (*n* = 1), polyarteritis nodosa (*n* = 1)

^e^Pemphigus vulgaris (*n* = 7), adult-onset Still disease (*n* = 6), bullous pemphigoid (*n* = 5), Behçet disease (*n* = 4), orbital pseudotumor (*n* = 3), unspecified inflammatory disorders (*n* = 3), autoinflammatory disorders (*n* = 2), cicatricial pemphigoid (*n* = 2), uveitis (*n* = 2), retroperitoneal fibrosis (*n* = 2), polymyalgia rheumatica (*n* = 1), aortitis (*n* = 1)

^f^Methotrexate (*n* = 60), adalimumab (*n* = 24), infliximab (*n* = 24), azathioprine (*n* = 23), mycophenolate mofetil (*n* = 22), mycophenolic acid (*n* = 3), tocilizumab (*n* = 19), leflunomide (*n* = 11), etanercept (*n* = 7), ustekinumab (*n* = 7), golimumab (*n* = 4), cyclophosphamide (*n* = 2), anakinra (*n* = 2), abatacept (*n* = 2), rituximab in the preceding 6 months (*n* = 2), chlorambucil (*n* = 1), ciclosporin A (*n* = 1), tacrolimus (*n* = 1), certolizumab (*n* = 1). More than one immunosuppressive agent was used in 37 patients (18 %)

^g^Hydroxychloroquine (*n* = 16), sulfasalazine (*n* = 7), colchicine (*n* = 2), dapsone (*n* = 2). More than one non-immunosuppressive DMARD was used in two patients

### Baseline serology

At baseline, 60 patients (30 %) were defined as seronegative for *S. pneumoniae*. Seronegativity was significantly associated with the use of systemic corticosteroids and lower total serum IgG levels (Table 1). Multivariate regression analysis confirmed the association of low levels of serum IgG against individual pneumococcal serotypes with systemic corticosteroids, as shown in Table 2. Other factors associated with a decrease in pneumococcus-specific IgG were age ≥65 years, active disease (PGA score ≥2) and low total serum Ig. In seronegative patients, infectious events had occurred during the preceding 3 months in 19 (32 %) of 60 compared with 18 (13 %) of 141 seropositives (*p* = 0.03) (Table 3).

**Table 2:** Multivariate regression analysis of factors associated with levels of serum IgG against pneumococcal serotypes 14, 19 and 23F in 201 patients at inclusion

	Serotype 14			Serotype 19			Serotype 23F		
	OR	95 % CI	*p*-Value	OR	95 % CI	*p*-Value	OR	95 % CI	*p*-Value
CTD vs. RA and SpA	2.33	1.15–4.71	0.019	1.11	0.68–1.81	NS	0.73	0.41–1.30	NS
Vasculitis vs. RA and SpA	1.30	0.59–2.87	NS	0.92	0.53–1.61	NS	0.84	0.44–1.61	NS
Other disease vs. RA and SpA	1.93	1.00–3.69	0.048	1.45	0.92–2.28	NS	1.62	0.97–2.71	0.067
Psoriasis vs. RA and SpA	1.60	0.91–2.81	NS	1.65	1.09–2.51	0.018	1.16	0.74–1.81	NS
Active disease^a^	–	–	NS	–	–	NS	0.67	0.46–0.97	0.035
Systemic corticosteroids	0.46	0.26–0.81	0.007	0.69	0.46–1.03	0.066	0.62	0.39–0.98	0.039
Older age^b^	–	–	NS	0.58	0.41–0.81	0.002	0.62	0.42–0.92	0.018
Low total serum IgA^c^	–	–	NS	–	–	NS	0.67	0.48–0.93	0.017
Low total serum IgM^d^	–	–	NS	0.77	0.57–1.04	NS	0.76	0.55–1.05	0.096

*Abbreviations*: *95 % CI* 95 % confidence interval, *CTD* connective tissue disease, *Ig* immunoglobulin, *NS* not significant, *OR* odds ratio, *RA* rheumatoid arthritis, *SpA* spondylarthritis. The variables used in this model were age at baseline serology, disease category; global disease activity according to the Physician Global Assessment score; use of systemic corticosteroids; use of immunosuppressive agents (cytotoxic drugs, methotrexate, tumor necrosis factor-α-blocking agents, anti-cytokine agents in general, monotherapy with biologic, or combination therapy); duration of immunosuppression; and total serum IgG, IgA and IgM

^a^Physician Global Assessment: very active and active versus low activity and inactive disease

^b^Age ≥65 yr versus <65 yr

^c^Serum IgA <2 g/L vs. ≥2 g/L

^d^Serum IgM <1 g/L vs. ≥1 g/L

**Table 3:** Infectious events and immunosuppressive treatment in 201 patients with inflammatory diseases, attributed to an observation and vaccination group according to pneumococcal serology at baseline

	Baseline			Follow-up		
Observed patients	Vaccination group^a^ (*n* = 60)	Observation group^b^ (*n* = 141)	All (*N* = 201)	Vaccination group^a^ (*n* = 56)	Observation group^b^ (*n* = 138)	All (*n* = 194)
Patients with infections, *n* (%)^c^	19 (32)	18 (13)	37 (18)	22 (39)	54 (39)	76 (39)
Infectious events, *n*	22	19	41	32	77	109
Localization/type of infection, *n* (%)						
Upper respiratory tract	10 (45)^d^	9 (47)	19 (46)	12 (38)	45 (58)	57 (52)
Lower respiratory tract	2 (9)	2 (11)	4 (10)	7 (22)	4 (5)	11 (10)
Urinary tract	3 (14)	1 (5)	4 (10)	5 (16)	16 (21)	21 (19)
Gastrointestinal	0 (0)	2 (11)	2 (5)	4 (13)	3 (4)	7 (6)
Shingles	1 (5)	0 (0)	1 (2)	1 (3)	3 (4)	4 (4)
Tuberculosis^e^	0 (0)	0 (0)	0	1 (3)	1 (1)	2 (2)
Skin infection	2 (9)	1 (5)	3 (7)	2 (6)	1 (1)	3 (3)
Other^f^ or not specified	4 (18)	4 (21)	8 (20)	0 (0)	4 (5)	4 (4)
Immunosuppressant, *n* (%)	50 (83)	131 (93)	181/201 (90)	38 (70)	118 (87)	156/190 (82)
Systemic corticosteroids, *n* (%)	41 (68)	50 (36)	91/201 (45)	28 (56)	43 (32)	71/185 (38)
Prednisone ≥10 mg/day at study start and end	–	–	–	5 (9)	12 (9)	17/194 (9)

^a^Immunoglobulin G (IgG) ≥0.5 μg/ml for fewer than four of six serotypes

^b^IgG ≥0.5 μg/ml for four or more of six serotypes

^c^Infectious events needing medical attention, at baseline, in the 3 months preceding inclusion and during the median (interquartile range) follow-up period of 384 (364–421) days

^d^Confirmed influenza A H1N1/09 in two patients, both of whom did not receive the pandemic influenza vaccine

^e^One case of reactivation of latent tuberculosis with intestinal involvement and one case of newly diagnosed latent tuberculosis

^f^Dental infection (*n* = 1), Chagas disease (*n* = 1), esophageal candidiasis (*n* = 1)

### Immunization with pneumococcal polysaccharide vaccine

**Fig. 2:**
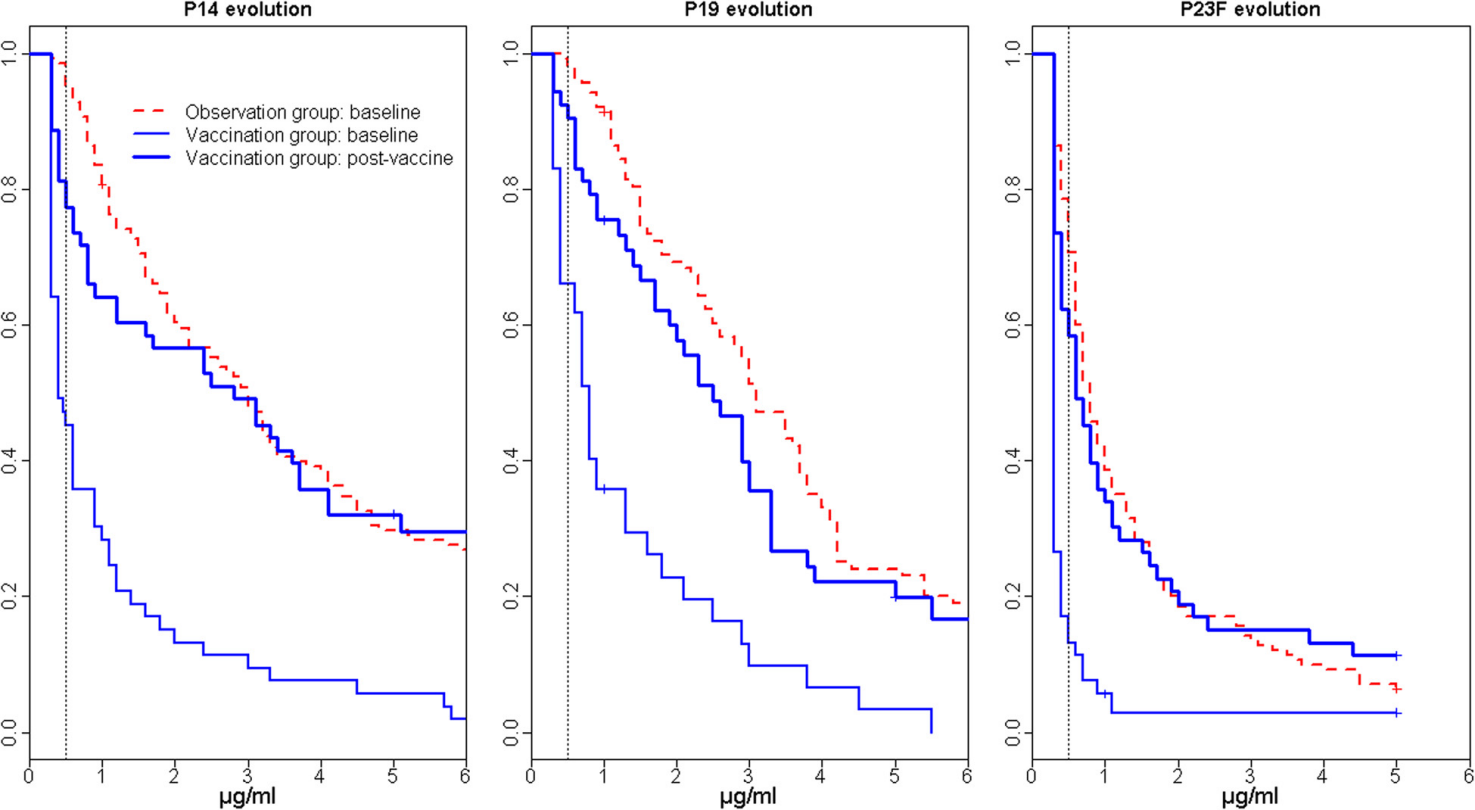
Serological response to vaccine serotypes 14, 19 and 23F at baseline and after 23-valent pneumococcal polysaccharide vaccine in the 53 immunized patients. Reverse cumulative distribution curves of specific serum immunoglobulin G to serotypes 14, 19 and 23F in the 53 immunized patients before 23-valent polysaccharide pneumococcal vaccine administration (*thin blue lines*) and 4–8 weeks after immunization (*bold blue lines*). Baseline serology of the 141 vaccine-naive patients in the observation group is shown for comparison (*dotted red lines*). Reverse cumulative distribution function takes into account censored values obtained by real-time enzyme-linked immunosorbent assay [28]

Fifty-seven patients received PPV. There was no systemic reaction to the vaccine. Mean disease activity according to the PGA score was 1.23 ± 0.61 before vaccination and 1.06 ± 0.69 1 month after immunization (*p* = NS). PGA scores did not change in 34 patients, decreased in 13 and increased in 6. Of the 53 patients assessed 4–8 weeks after PPV, 46 (87 %) reached seropositive thresholds (Fig. 2). Non-responders had lower IgM levels (median IgM of 0.66 g/L compared with 0.9 g/L in responders; *p* = 0.044) and tended to have higher corticosteroid dosages (daily prednisone equivalent ≥20 mg in 57 % compared with 22 % of responders; *p* = 0.07).

### One-year follow-up

**Fig. 3:**
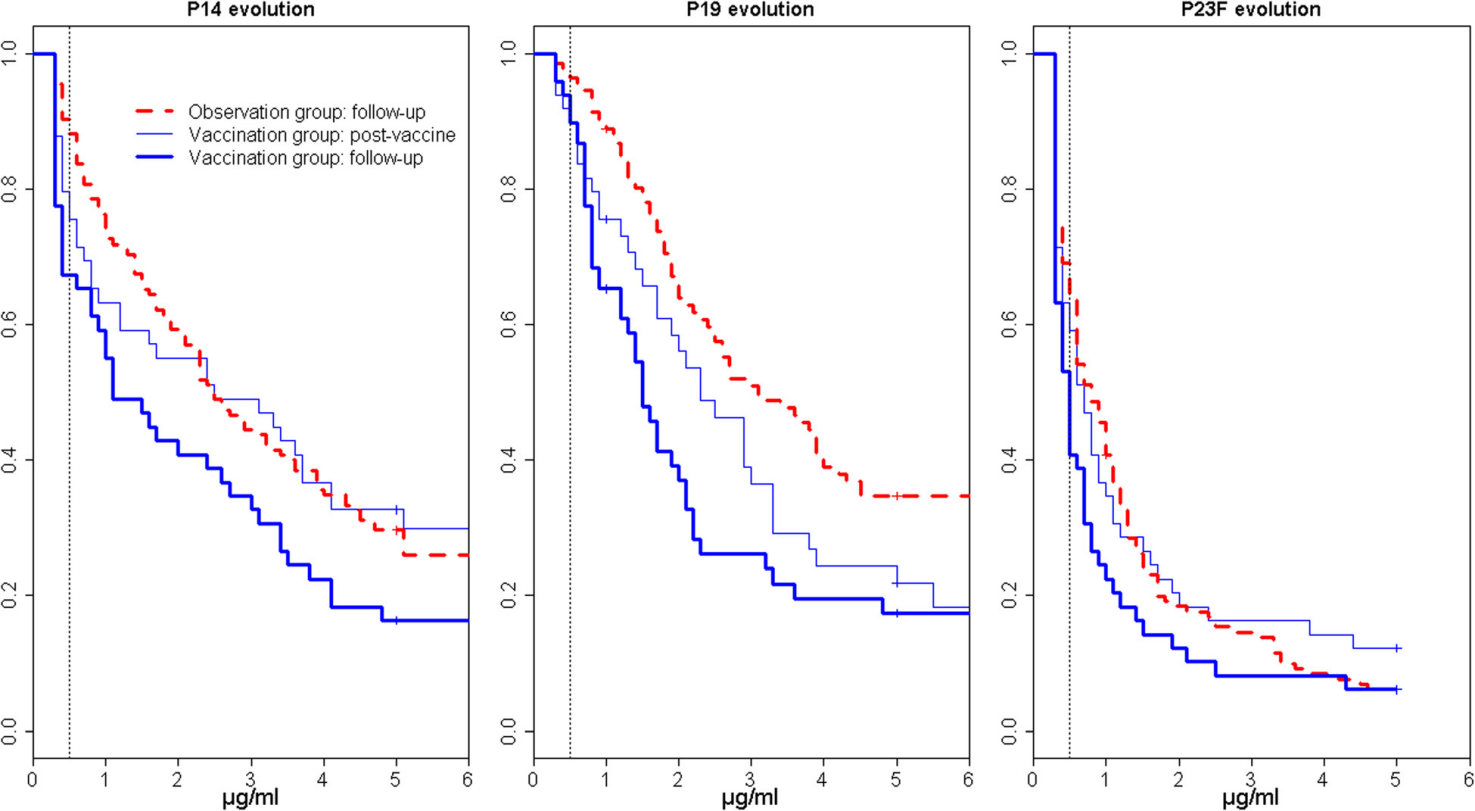
Evolution of serology to vaccine serotypes 14, 19 and 23F in 49 patients immunized with the 23-valent polysaccharide pneumococcal vaccine compared with 135 patients observed after 4–8 weeks and after 1 year. Reverse cumulative distribution curves of specific serum immunoglobulin G (IgG) against serotypes 14, 19 and 23F in patients immunized 4–8 weeks after vaccine (*thin blue lines*) and after 1 year (*bold blue lines*) compared with patients observed after 1 year (*dotted red lines*). Reverse cumulative distribution function takes into account censored values obtained by real-time enzyme-linked immunosorbent assay [28]. Median (95 % confidence interval) specific IgG titers to pneumococcal serotype 14 decreased from 2.5 (1.2–4.1) μg/ml 4–8 weeks after immunization to 1.1 (0.8–3.0) μg/ml after 1 year (*p* = 0.001 by from paired Prentice–Wilcoxon test for censored paired data), to serotype 19 from 2.3 (1.7–3.3) to 1.5 (1.2–2.1) μg/ml (*p* = 0.009) and to serotype 23F from 0.7 (0.5–1.1) to 0.5 (0.4–0.7) μg/ml (*p* = 0.005). In patients observed, median (95 % confidence interval) IgG values after 1 year were 2.5 (2.1–3.4) μg/ml to serotype 14, 3.1 (2.5–4.2) μg/ml to serotype 19 and 0.8 (0.6–1.0) to serotype 23F

Disease activity according to PGA score decreased from 1.12±0.77 at baseline to 0.93±0.76 (*p* = 0.004) in the observation group and from 1.2±0.56 to 0.91±0.88 (*p* = 0.025) in the vaccination group. Follow-up serology was performed within a median (IQR) of 383 (364–421) days in patients observed and 369 (342–392) days in patients vaccinated. Within the observation group, 13 (10 %) of 135 had become seronegative to *S. pneumoniae* compared with 16 (33 %) of 49 immunized patients (*p* < 0.001). Of the 42 patients seropositive after PPV, 9 (21 %) had become seronegative. The decline in antibody titers to serotypes 14, 19 and 23F after 1 year in vaccinated patients compared with those observed is shown in Fig. 3. Of the 16 seronegative patients 1 year after PPV, 12 (75 %) were still taking corticosteroids. Four of them had prednisone doses ≥10 mg/day both at inclusion and at the end of follow-up. In comparison, 16 (53 %) of the seropositive patients were treated with corticosteroids, with 2 having prednisone doses ≥10 mg/day at both study start and end (*p* = 0.086). There was no difference regarding the type of immunosuppressant used.

During follow-up, 109 infectious events were reported in 54 (39 %) of 138 patients observed and in 22 (39 %) of 56 of those in the vaccination group (Table 3). There was no documented pneumococcal infection and no event suggestive of IPD. Severe infections included one case of intestinal tuberculosis and one case of legionellosis, both in patients treated with TNF-α-blocking agents (one in the observation group and the other in the vaccination group).

When taking into account pneumococcal serology at the end of follow-up, regardless of immunization, infections had occurred in 54 (34.4 %) of 157 seropositive patients compared with 19 (59.4 %) of 32 in seronegative patients (*p* = 0.008). In patients who received PPV, overall infections occurred in 12 (36 %) of 34 of those seropositive, compared with 9 (60 %) of those seronegative, at 1 year (*p* = NS). Infections of the airways potentially prevented by the vaccine were reported in 8 patients (24 %) seropositive, compared with 8 patients (50 %) who were seronegative, 1 year after PPV (*p* = 0.07).

Infections occurred in 14 (82 %) of 17 patients taking prednisone ≥10 mg/day at both study start and end, compared with 62 (35 %) of 122 patients without sustained high-dose prednisone or without corticosteroids (*p* < 0.001). Besides regular prednisone intake of ≥10 mg/day, no other predictors of infections were found (Table 4).

**Table 4:** Predictors of reported infections during the 1-year follow-period in 194 patients treated with immunosuppressive drugs for an inflammatory disease

	Overall infections			Infections of the airways		
	RR	95 % CI	*p*-Value	RR	95 % CI	*p*-Value
Sustained systemic corticosteroids^a^	2.35	1.75–3.17	<0.001	2.43	1.56–3.80	<0.001
Active disease^b^	1.13	0.77–1.66	0.534	1.51	0.97–2.35	0.069
Age at inclusion ≥65 yr vs. <65 yr	0.99	0.66–1.48	0.947	0.87	0.51–1.48	0.604

*Abbreviations*: *95 % CI* 95 % confidence interval, *RR* univariate relative risk

^a^Prednisone equivalent ≥10 mg/day at inclusion and at the end of follow-up vs. <10 mg/day

^b^Physician Global Assessment score 2 and 3 (active and very active disease) vs. 0 and 1 (inactive or moderately active disease) at inclusion

## Discussion

Pneumococcal vaccines are infrequently administered to patients with immune-mediated inflammatory disorders, despite an associated increased risk of pneumonia and IPD. This is likely due to concerns about vaccine safety and efficacy. Recommendations regarding pneumococcal vaccination rely on weak evidence, and there are doubts about the efficacy of polysaccharide vaccines in adults undergoing immunosuppressive treatment. In this prospective study, we recruited 201 adults with immune-mediated inflammatory disorders undergoing immunosuppressive treatment, to whom no pneumococcal vaccine had been administered in the previous 5 years. Most if not all of these patients had never been immunized against *S. pneumoniae*. Less than 60 % of patients had received influenza vaccines, despite these vaccines’ being actively promoted in this risk group, confirming the low compliance with vaccine guidelines.

We found that 70 % of patients had antibodies to the six *S. pneumoniae* serotypes assessed. Seronegative patients had more active disease and accordingly were more often treated with systemic corticosteroids. They also had lower total serum IgG levels. Patients with systemic vasculitis were more often seronegative, in contrast to those with psoriasis. This finding is probably due to predominant use of corticosteroids and cytotoxic drugs in vasculitis patients, compared with patients with psoriasis and arthritis, who were frequently treated with TNF-α-blocking agents alone. Others have assessed prevaccination antibody levels to *S. pneumoniae* in patients with RA and SLE without noticing significant differences according to disease or immunosuppressive treatment [30, 31]. We also found significantly lower prevaccination antibody titers to serotypes 19 and 23F in patients older than 65 years of age. Other studies have addressed serological responses in the elderly, which has led to recommendations to give persons over 65 years of age PPV [12, 13].

PPV was well tolerated, with no reports of systemic reaction. Only a few patients had an increase in disease activity after vaccination. Other prospective studies, including our previous work on a similar population, have shown that vaccines does not usually trigger disease flares in patients with chronic inflammatory diseases [11, 23, 24, 32, 33].

Serological responses assessed shortly after PPV were good, with 87 % of the vaccinated patients reaching seropositive thresholds. Non-responders had higher corticosteroid doses. Others have linked diverse immunosuppressants to reduced vaccine response. Elkayam et al. showed that in patients with RA and SLE treated with various disease-modifying antirheumatic drugs, up to one-third of patients vaccinated with PPV had an insufficient response: 14 (33.3 %) of 43 patients with RA and 5 (20.8 %) of 24 patients with SLE responded to 0 or 1 of 7 vaccine serotypes assessed [30]. In particular, combination treatment with methotrexate and TNF-α-blocking agents resulted in poor vaccine responses [23, 31, 34–36], whereas this was not seen in patients treated solely with TNF-α-blocking agents [24, 31, 34, 37, 38]. In our patients, methotrexate was not associated with poor response to PPV, nor were there other predictors of short-term vaccine response besides corticosteroids, which is possibly due to the small number of immunized patients in our sample. In healthy individuals, antibody levels toward PPV serotypes remain high for at least 5 to 10 years postvaccination [39]. A small study on 19 patients with SLE showed a decrease in antibody levels below the protective level in 42 % of patients 3 years after PPV [20]. A more rapid decline was shown in patients with RA undergoing immunosuppressive treatment [21]. In our study, 90 % of the initially seropositive patients maintained specific antibody titers over the course of 1 year without vaccination. In contrast, those patients initially seronegative and who responded to PPV had a significant decrease in antibody titers over the next year. This rapid loss of serological response was also associated with corticosteroids.

There were no differences in the rate of clinical infections in patients vaccinated and observed during follow-up. No pneumococcal infection was documented. Overall, infectious events were, however, associated with seronegativity. This suggests that pneumococcal serology is a marker of the degree of immunosuppression in our population, with those treated with high-dose corticosteroids having lower titers of pneumococcal IgG. Sustained corticotherapy was also associated with an increased rate of infection, as shown by others [5, 25]. This poses a clinical dilemma. On the one hand, although PPV seems safe in patients with active disease, concomitant treatment with corticosteroids may hamper vaccine response. On the other hand, these patients are also at greatest risk for infections. With the serological results shown, one would be tempted to postpone PPV until corticosteroids have been lowered. However, those who stayed seropositive 1 year after PPV tended to have fewer respiratory infections than seronegative patients (*p* = 0.07). Thus, some of these patients may benefit from PPV in the short term through a reduction of non-invasive pneumococcal infections. Conjugated pneumococcal vaccines are currently being studied in adults with immunosuppression and may increase the likelihood of sustained serological response.

One of the greatest difficulties in the evaluation of vaccine response is to determine whether serum antibody titers correlate with clinical protection. Functional antibodies measured by opsonophagocytosis may be more relevant for protection against pneumococcal infections than antibodies assessed by ELISA. Limited resources did not allow us to include either this assay or the analysis of antibodies to more than six serotypes. Another possible limitation of the present study is our definition of serological response. The WHO considers levels of IgG antibodies to individual pneumococcal serotypes of 0.35 μg/ml or greater as protective against IPD [40]. However, serotype-specific antibody levels correlating with protection differ for each serotype and each clinical endpoint, as well as in various populations. Healthy individuals are expected to respond to at least half of the evaluated serotypes following vaccination [37]. We therefore chose to define seropositivity and vaccine response as a specific IgG level greater than or equal to 0.5 μg/ml to at least four of the six tested serotypes, being aware that these are empirical criteria. The absence of documented pneumococcal infections may be seen as another limitation of the study, but the expected incidence of pneumococcal disease in this population was low, particularly over the observation period of 1 year. In addition, pneumococcal infections can be somewhat difficult to prove, as cultures or urinary pneumococcal antigen testing are not always performed.

The strength of our study lies in the combination of serologies and clinical assessment, in particular regarding disease activity, immunosuppressive treatment and infectious complications. Patients were prospectively followed, and only a few were lost to follow-up by the end of the study. We did not randomize patients at inclusion to receive PPV, which would have been unethical. Only seronegative patients received the vaccine, which we thought would be those most in need of protection. The heterogeneity in immune-mediated inflammatory disorders and treatments may be seen as a limitation, but it reflects a real-life setting.

## Conclusions

This study underlines the good tolerance of pneumococcal immunization in immunosuppressed adults with immune-mediated inflammatory diseases, as well as the limited immunogenicity of PPV, which elicits very transient responses. In our patients, low serum IgG to pneumococcal antigens reflected the severity of the underlying disease and the degree of immunosuppression. Systemic corticosteroids in particular were associated with poor serological response and an increase in various infectious events. This study adds evidence to the poor performance of PPV in adults on immunosuppressive medication and should motivate the development of newer vaccine strategies in this particular setting. Studies are needed to assess whether the conjugate vaccine is well tolerated and leads to effective protection from pneumococcal invasive disease in this fragile population.

## Abbreviations

CTD: connective tissue disease
DMARD: disease-modifying antirheumatic drug
ELISA: enzyme-linked immunosorbent assay
Ig: immunoglobulin
IPD: invasive pneumococcal disease
IQR: interquartile range
NS: not significant
PGA: Physician Global Assessment
PPV: pneumococcal polysaccharide vaccine
RA: rheumatoid arthritis
SLE: systemic lupus erythematosus
SpA: spondylarthritis
TNF-α: tumor necrosis factor α
WHO: World Health Organization
